# Zoonosis screening in Spanish immunocompromised children and their pets

**DOI:** 10.3389/fvets.2024.1425870

**Published:** 2024-07-23

**Authors:** Paula Garcia-Sanchez, David Romero-Trancón, Iker Falces-Romero, Paula Navarro Carrera, Guillermo Ruiz-Carrascoso, David Carmena, María Casares Jiménez, Antonio Rivero-Juárez, Laura Moya, Jaume Rodón, Fernando Esperón, Belén Pérez-Hernando, Rocío Sánchez-León, Jara Hurtado-Gallego, Sonia Alcolea, Talía Sainz, Cristina Calvo, Ana Méndez-Echevarría

**Affiliations:** ^1^Pediatric Emergency Department, La Paz University Hospital, Madrid, Spain; ^2^Hospital La Paz Institute for Health Research (IdiPAZ), Madrid, Spain; ^3^Doctoral Program in Medicine and Surgery, Autonomous University of Madrid, Madrid, Spain; ^4^Clinical Microbiology Department, La Paz University Hospital, Madrid, Spain; ^5^Center for Biomedical Research in Infectious Diseases Network (CIBERINFEC), Carlos III Health Institute, Madrid, Spain; ^6^Parasitology Reference and Research Laboratory, National Center of Microbiology, Carlos III Health Institute, Majadahonda, Spain; ^7^Infectious Diseases Unit, Reina Sofía University Hospital, Maimónides Biomedical Research Institute of Córdoba, University of Córdoba, Córdoba, Spain; ^8^Idexx Laboratories, Barcelona, Spain; ^9^Veterinary Faculty, European University of Madrid, Villaviciosa de Odón, Madrid, Spain; ^10^Doctoral Program in Microbiology, Autonomous University of Madrid, Madrid, Spain; ^11^Pediatric Infectious and Tropical Diseases Department, La Paz University Hospital, Madrid, Spain; ^12^Pediatrics Department, Severo Ochoa Leganés University Hospital, Madrid, Spain; ^13^Puerta de Hierro Health Research Institute (IDIPHISA), Majadahonda, Spain; ^14^Pediatric Department, Autonomous University of Madrid, Madrid, Spain; ^15^Translational Research Network in Pediatric Infectious Diseases, Madrid, Spain; ^16^ERN TransplantChild, Madrid, Spain

**Keywords:** children, colonization, emerging pathogens, immunocompromised, infection, pets, zoonoses

## Abstract

**Introduction:**

Although pets provide several social–emotional benefits for children, the risk of zoonosis must be considered among immunocompromised individuals.

**Methods:**

A prospective study was conducted in a tertiary hospital including immunocompromised patients younger than 20 years owning dogs and/or cats. Colonization and/or infection was evaluated by stool studies, bacterial swabs, blood polymerase chain reaction and serological studies in both patients and their pets, to evaluate potential zoonotic transmission occurrence.

**Results:**

We included 74 patients and their 92 pets (63 dogs, 29 cats). Up to 44.6% of the patients and 31.5% of the pets had at least 1 positive result. Up to 18.4% of pets’ fecal samples were positive (bacteria, parasites or hepatitis E virus). No helminths were observed despite the high frequency of incorrect intestinal deworming practices. Among children, gastrointestinal microorganisms were found in 37.3% (primarily *Clostridium difficile*). Colonization by *Staphylococcus pseudintermedius* was common among pets (8.0%) but not among children (0.0%). No shared colonization between owners and pets was observed, except in one case (*Blastocystis* in both patient and pet feces). Among patients, serologies were positive for *Strongyloides stercoralis* (14.8%), *Toxocara canis* (3.2%), *Bartonella henselae* (19.1%) and hepatitis E (5.6%). Serology was positive for *Rickettsia* spp. (22.6%) and *Babesia* spp. (6.5%) in dogs and for *Leishmania* spp. (14.3%) and *Toxoplasma* spp. (14.3%) in cats.

**Conclusion:**

Exposure to zoonotic agents was detected in both patients and pets; however, shared colonization events were almost nonexistent. In our cohort, dogs and cats do not appear to entail high zoonosis transmission risk for immunocompromised patients.

## Introduction

Pets play an important role in the social–emotional development of children (1) and contribute to a healthier lifestyle. Interaction with animals can have additional positive effects in patients with chronic medical conditions (1). However, animal contact can also imply zoonotic risks, particularly for immunocompromised children (2, 3).

Case reports of viral, bacterial and parasitic zoonotic agents transmitted from pets to immunocompromised patients can be found in the literature (4–6) and immunocompromised patients who live with pets are asked to take special precautions (2). However, the moderate to poor evidence of most recommendations, together with the insufficient knowledge or awareness of zoonotic diseases among both patients and healthcare professionals often leads to gaps in the fulfillment of preventive measures (7–9). Low compliance with deworming protocols and failure to comply with pets’ immunization schemes have been reported by our and other groups in previous studies (3, 7, 9, 10).

In recent years, the role of some emerging zoonotic pathogens, such as hepatitis E virus (HEV) or *Enterocytozoon bieneusi*, are gaining increasing relevance, although the transmission routes between humans and animals remain uncertain (11, 12).

In addition to the infection risk, household pets can be colonized by bacteria that produce human diseases‚ such as *Staphylococcus aureus*, *Staphylococcus pseudintermedius*, multidrug-resistant *Enterobacteriaceae*, or *Clostridium difficile* (13–16), hypothetically increasing the risk of colonization of cohabiting children.

Few studies have evaluated the presence of zoonotic agents among immunocompromised patients and their pets and, to date, the evidence is insufficient to quantify the real risk of immunocompromised children acquiring a zoonotic infection from their pets (2, 3). A One Health approach is very much needed on the basis of collaboration between human and veterinary medicine (17). Both the number of immunocompromised patients and pet ownership have increased exponentially in recent decades (7, 18). We aimed to determine the prevalence of colonization and/or infection by microorganisms that can cause zoonoses in immunocompromised children and their pets and identify potential risk factors for colonization/infection.

## Materials and methods

A prospective study was performed from July 2022 to April 2024 at La Paz Pediatric University Hospital, a large tertiary hospital in Madrid, Spain, and a National Reference Center for immunocompromised children. Pediatric infectious disease specialists conducted the study, in collaboration with veterinarians and microbiologists. The study was approved by the local Clinical Research Ethics Committee of La Paz University Hospital (PI-4770) and all participants and/or legal guardians provided informed consent.

All immunocompromised patients under medical follow-up in our hospital younger than 20 years of age were invited to participate in the study if they owned at least 1 pet (dog and/or cat). We considered the following immunocompromised patients:

- Patients who had received solid organ transplantation in the previous 10 years.
- Patients who had received hematopoietic stem cell transplantation in the last 5 years, or in the last 5–10 years if the immune reconstitution was incomplete and/or required immunosuppressive treatment at the time of the study.
- Patients who had been diagnosed with genetically confirmed inborn errors of immunity.
- Patients with oncological diseases undergoing chemotherapy.
- Patients with rheumatological diseases who were receiving immunosuppressive treatments.

Patients (and/or families) who fulfilled the inclusion criteria were contacted by telephone or in person during hospital appointments. Those owning a dog and/or a cat and willing to provide informed consent for participation completed 2 standardized questionnaires: one document containing the patient’s information (sociodemographic data and basic medical data) and a second document with information regarding the pet(s), including type of pet, veterinary care, patient’s interaction with the animal, vaccines, anti-parasitic treatments, previous illnesses and dietary information. The questionnaires were completed during a hospital visit or online (Supplementary File 1). Patients aged 12 years or older completed the questionnaire themselves, whereas in the case of children younger than 12, the parents were asked to complete it. Relevant clinical data were obtained by a pediatrician member of the study team reviewing the patient’s medical records. A veterinarian reviewed the aspects related to animal health and care and, when necessary, the pet’s veterinarian was contacted by phone.

Screening for potentially animal-transmitted infections and colonizations was conducted in patients and pets. Stool culture and multiplex polymerase chain reaction (PCR) for fecal pathogens were performed, including *Aeromonas* spp., *Campylobacter* spp., *Clostridium difficile* toxin B, S*almonella* spp., S*higella* spp., *Escherichia coli*, *Vibrio* spp., *Yersinia enterocolitica,* C*yclospora cayetanensis, Dientamoeba fragilis*, *Entamoeba histolytica*, *Giardia duodenalis*, *Cryptosporidium* spp., *Blastocystis* sp., *Enterocytozoon bieneusi* and *Encephalitozoon* spp. The presence of helminths was also analyzed in pets’ feces (*Ascaris* spp., Ancylostomatidae, Trichuridae and Cestoda). Nasopharyngeal and rectal swabs for screening of colonization by resistant bacteria and serological studies for the most common zoonotic agents were also performed in both patients and pets. Patients undergoing immunoglobulin treatment or with treatments affecting antibody production were excluded from the serological study. PCR assays for the diagnosis of acute HEV infection were performed in patients’/pets’ feces, blood and sera. Table 1 summarizes the main microbiological tests performed in patients and pets. Supplementary File 2 details the main microbiological techniques used.

**Table 1:** Microbiological tests performed in patients and pets.

Patients		
Stool culture and multiplex polymerase chain reaction (PCR) for fecal pathogens	Bacterial culture	PCR
	*Aeromonas* spp., *Campylobacter* spp., *Clostridium difficile* toxin B, S*almonella* spp., S*higella* spp., Enteroinvasive *Escherichia coli*, *Vibrio* spp., and *Yersinia enterocolitica.*	Parasites: *Blastocystis* sp., *Cryptosporidium* spp., C*yclospora cayetanensis, Dientamoeba fragilis*, *Encephalitozoon* spp., *Entamoeba histolytica, Enterocytozoon bieneusi,* and *Giardia duodenalis.* Viruses*: Paslahepevirus* and *Rocahepevirus.*
Nasopharyngeal swab for screening colonization by resistant bacteria	Methicillin-resistant *Staphylococcus aureus*, *S. pseudintermedius*	
Rectal swab for screening colonization by resistant bacteria	Extended-spectrum beta-lactamase-producing *Enterobacteriaceae*, and carbapenem-resistant bacteria	
Serology	*Toxocara canis* and *Strongyloides stercoralis* (dog owners); *Toxoplasma gondii* and *Bartonella henselae* (cat owners); Hepatitis E virus, SARS-CoV-2 (dog and/or cat owners).	
Blood PCR	Hepatitis E virus	
PETS		
Stool culture and multiplex polymerase chain reaction (PCR) for fecal pathogens.	Bacterial culture	PCR
	*Aeromonas* spp., *Campylobacter* spp., *Clostridium difficile* toxin B, S*almonella* spp., S*higella* spp., Enteroinvasive *Escherichia coli*, *Vibrio* spp., and *Yersinia enterocolitica.*	Parasites: *Blastocystis* sp., *Cryptosporidium* spp., C*yclospora cayetanensis, Dientamoeba fragilis*, *Encephalitozoon* spp., *Entamoeba histolytica, Enterocytozoon bieneusi*, and *Giardia duodenalis.* Viruses*: Paslahepevirus* and *Rocahepevirus.*
Antigen detection and microscopic diagnosis in feces	*Giardia duodenalis*, *Ascaris* spp., Ancylostomatidae, Trichuridae, and Cestoda	
Nasopharyngeal swab for screening colonization by resistant bacteria	Methicillin-resistant *Staphylococcus aureus*, *S. pseudintermedius*	
Rectal swabs for screening colonization by resistant bacteria	Extended-spectrum beta-lactamase-producing *Enterobacteriaceae*, and carbapenem-resistant bacteria	
Serology	Dogs	Cats
	*Leishmania* spp.*; Borrelia burgdorferi; Rickettsia* spp.; *Ehrlichia canis; Babesia canis; Anaplasma* spp.; *Leptospira*	*Toxoplasma gondii; Leptospira; Leishmania* spp.
Blood PCR	Hepatitis E virus	
PCR in blood and urine	*Leptospira* (in case of a positive serology)	

Stool samples from patients and pets were collected by their families. Patients’ swabs and blood samples were collected during scheduled hospital appointments. Pets’ swabs were collected by their owners following an explanatory sheet created for this purpose and their blood extraction was performed in veterinary clinics.

### Statistical analysis

The statistical analysis was performed using the Statistical Package for the Social Sciences (IBM SPSS Statistics Version 21, IBM Inc., Chicago, IL, USA). Qualitative data were presented as absolute frequencies and percentages and quantitative variables were expressed as the main measures of centralization and dispersion (mean, standard deviation, median, minimum, maximum, interquartile range [IQR]).

For the study of risk factors for colonization by zoonotic agents, a univariate analysis was performed. Pearson’s chi-squared test (or Fisher’s exact test for 2×2 tables or likelihood ratio in mXn tables, if necessary) was used for qualitative variables; *p*-values under 0.05 were considered significant.

## Results

### Characteristics of study participants

**Figure 1:**
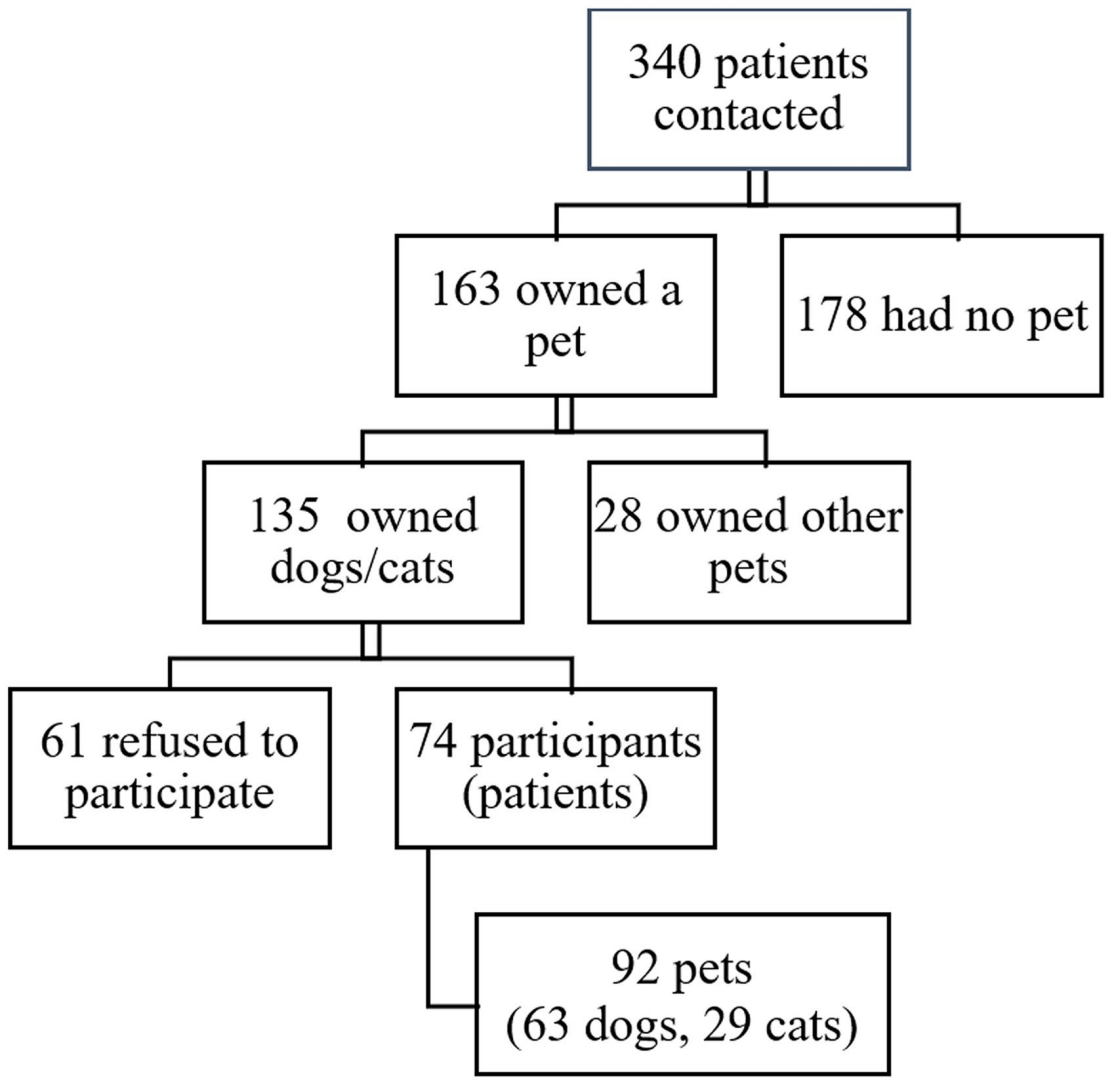
Flow chart of contacted and participating patients and pets.

A total of 340 immunocompromised patients were contacted, 163 (47.9%) of whom owned a pet, mainly dogs and/or cats (135; 82.8%). Ultimately, 74 patients (51.3% female, median age 10.2 years [IQR 6.8–13.8]) and their 92 pets (63 dogs and 29 cats) were included in the study (Figure 1).

Table 2 summarizes the clinical characteristics of the included patients and pets and Table 3 shows the pets’ main data, feeding and veterinary care (2, 19–22). Most (86.4%) owners took their pets to the veterinarian at least once a year and 96.5% of the pets were fed with commercially processed food. Up to 84.4% of newly acquired pets were puppies or kittens. Although 92.2% of the pets underwent intestinal deworming, only 4.9% underwent it monthly and 23.4% of owners reported having found ticks on their pets.

**Table 2:** Clinical characteristics of the included patients and pets.

Characteristic	*n* (%)
Male	36 (48.6)
Median age in years (IQR)	10.2 (6.8–13.8)
Median age since transplantation (IQR)	4 (1.8–6.9)
Underlying disease	
Solid organ transplantation	44 (59.5)
Kidney	19 (25.7)
Liver	13 (17.6)
Heart	7 (9.5)
Multivisceral	4 (5.4)
Lung	1 (1.3)
Hematopoietic stem-cell transplantation	14 (18.9)
Inborn errors of immunity	4 (5.4)
Oncological diseases	6 (8.1)
Rheumatological diseases	6 (8.1)
Opinion on pet ownership	
Benefit	57 (77.0)
Risk	8 (10.8)
Did not answer	9 (12.2)
Type of pet included	
Dog	63 (68.5)
Cat	29 (31.5)

**Table 3:** Data on hygiene, feeding, and veterinary care of pets, and patients’ attitudes.

Age of the pet at acquisition	<6 months		**84.4%** (76/90)
	6 months – 1 year		4.4% (4/90)
	1 year – 5 years		5.5% (5/90)
	>5 years		6.7% (6/90)
Veterinarian visits	≥3 times/year		28.4% (25/88)
	2 times/year		17.0% (15/88)
	1 time/year		40.9% (36/88)
	<1 time/year		**13.6%** (12/88)
Internal deworming	Yes		92.2% (83/90)
	Monthly		4.9% (4/82)
	Every 3 months		48.8% (40/82)
	Every 6 months		23.2% (19/82)
	Sporadically		**23.2%** (19/82)
	No		**7.8%** (7/90)
External deworming	Yes		73.9% (65/88)
	Monthly		18.7% (12/64)
	Every 3 months		23.4% (15/64)
	Every 6 months		39.1% (25/64)
	Sporadically		**18.7%** (12/64)
	No		**26.1%** (23/88)
Animal feeding	Commercial processed food		96.5% (84/87)
	Home cooked food		**2.3%** (2/87)
	Undercooked or raw food		**1.1%** (1/87)
Pet outdoors	Yes		**54%** (47/87)
	Daily		82.6% (38/46)
	1 time/week		10.9% (5/46)
	Monthly		4.3% (2/46)
	Every 6 months		2.2% (1/46)
	1 time/year		0.0% (0/46)
	No		46.0% (40/87)
Hunting	Yes		**17.0%** (15/88)
	No		83% (73/88)
Ticks on the pet	Yes		**23.9%** (21/88)
	No		76.1% (67/88)
Previous infectious diseases in the pet	Yes		**1.1%** (1/89)
	No		98.9% (88/89)
Vaccination	Rabies	Yes	74.7% (68/91)	
		No	**23.1%** (21/91)
		Unknown	2.2% (2/91)
	*Bordetella bronchiseptica*	Yes	14.9% (13/87)	
		Nasal	**30.8%** (4/13)
		Oral	**7.7%** (1/13)
		Injectable	46.2% (6/13)
		Unknown	15.4% (2/13)		
		No	**65.5%** (57/87)
		Unknown	19.5% (17/87)
	*Leptospira* (dogs)	Yes	51.5% (34/66)
		No	**7.6%** (5/66)
		Unknown	40.9% (27/66)
*Leishmania* protection (dogs)	Vaccination	Yes	22.2% (14/63)
		No	**77.8%** (49/63)
	Collar	Yes	39.7% (25/63)
		No	**60.3%** (38/63)
	Pipette	Yes	39.7% (25/63)
		No	**39.7%** (38/63)

^*^Patients who did not comply with the veterinary recommendations of the guidelines for immunocompromised patients with pets or who had risk attitudes for acquiring zoonoses are shown in bold (2, 19–22).

Regarding the risk–benefit balance of pet ownership, 77.0% (57/74) of the respondents believed that the benefits of pet ownership outweighed the risks, whereas 10.8% (8/74) thought that pet ownership was more risky than beneficial and 12.2% (9/74) did not answer this question.

### Microbiological results

Although not all samples were available from all participants and their pets, 33 (44.6%) patients had at least one positive result in the tests performed, including bacterial swabs (4.6%, 3/65), fecal samples (37.3%, 22/59) and blood serologies (22.5%, 16/71). Almost one-third of the pets (31.5%, 29/92) had positive results: 8.1% of nasopharyngeal swabs (7/86), 18.4% of fecal samples (16/87) and 26.3% of blood serologies (10/38). However, only one case of shared colonization involving *Blastocystis* was identified in stool samples (ST4 in a patient, unknown subtype in a dog) and no zoonotic transmission event could be demonstrated. No helminths were found in the stool tests of any pet, despite the presence of a high frequency of incorrect intestinal deworming regimens.

Specific results from nasopharyngeal and rectal bacterial swabs and from stool samples are summarized in Table 4, including the total number of samples in each category. Colonization by *S. pseudintermedius* was more common among pets (8%), compared to patients (0%). Up to 18.4% of pet fecal samples were positive, with the following microbiological findings: *Cryptosporidium* spp. (4.6%), *E. bieneusi* (3.4%), *Campylobacter* spp. (3.4%), *G. duodenalis* (2.3%), hepatitis E virus (2.3%), *D. fragilis* (1.1%), *Blastocystis* sp. (1.1%) and *Encephalocytozoon* spp. (1.1%). Among children, gastrointestinal microorganisms were found in 37.3% (primarily *C. difficile*, followed by *Blastocystis* sp. (6.8%), *G. duodenalis* (5.1%), *D. fragilis* (3.4%), hepatitis E virus (3.4%), *Campylobacter* spp. (1.7%), *Y. enterocolitica* (1.7%), *Aeromonas* spp. (1.7%) and *Cryptosporidium* spp. (1.7%). Results from serology and blood PCR are summarized in Table 5. Among patients, serological tests were positive for *Strongyloides stercoralis* (14.8%), *Toxocara canis* (3.2%), *Bartonella henselae* (19.1%) and hepatitis E (5.6%). In dogs, serologies were positive for *Rickettsia* spp. (22.6%) and *Babesia canis* (6.5%). One cat tested positive for *Leishmania* spp. and another cat tested positive for *Toxoplasma* spp. Supplementary File 3 summarizes the main molecular findings and sequencing data from pathogens found in feces.

**Table 4:** Results from nasopharyngeal and rectal swabs and fecal samples.

Nasopharyngeal and rectal swabs					
	Patients (*n* = 65)	Pets (*n* = 86)			% Shared colonization
		Dogs (*n* = 58)	Cats (*n* = 28)	Global	
*Staphylococcus aureus* (NFS)	0 (0.0%)	0 (0.0%)	0 (0.0%)	0 (0.0%)	0.0%
*Staphylococcus pseudintermedius* (NFS)	0 (0.0%)	**7 (12.1%)**	0 (0.0%)	**7 (8.1%)**	
Resistant *enterobacteriaceae* (RS)	3 (4.6%)	0 (0.0%)	0 (0.0%)	0 (0.0%)	
Feces					
	Patients(*n* = 59)	Pets (*n* = 87)			% Shared colonization
		Dogs (61)	Cats (26)	Global	
Bacteria					Only in one case was detected the same pathogen in a pet and its owner (*Blastocystis*). ST4 in a patient, unknown subtype in a dog (subtyping was not possible due to a high C_T_ value).
*Clostridium difficile*	11 (18.6%)*	0 (0.0%)	0 (0.0%)	0 (0.0%)	
*Campylobacter* spp.	1 (1.7%)	2 (3.3%)	1 (3.8%)	3 (3.4%)	
*Yersinia enterocolitica*	1 (1.7%)	0 (0.0%)	0 (0.0%)	0 (0.0%)	
*Aeromonas* spp.	1 (1.7%)	0 (0.0%)	0 (0.0%)	0 (0.0%)	
Parasites					
*Blastocystis* spp.	4 (6.8%)	1 (1.6%)	0 (0.0%)	1 (1.1%)	
*Giardia duodenalis*	3 (5.1%)	1 (1.6%)	1 (3.8%)	2 (2.3%)	
*Dientamoeba fragilis*	2 (3.4%)	1 (1.6%)	0 (0.0%)	1 (1.1%)	
*Cryptosporidium* spp.	1 (1.4%)	3 (3.3%)	1 (3.8%)	4 (4.6%)	
*Enterocytozoon bieneusi*	0 (0.0%)	3 (3.3%)	0 (0.0%)	3 (3.4%)	
*Encephalitozoon* sp.	0 (0.0%)	0 (0.0%)	1 (3.8%)	1 (1.1%)	
Viruses					
*Hepevirus*	2 (3.4%)	1 (1.6%)	1 (3.8%)	2 (2.3%)	
Helminths	Not done	0 (0.0%)	0 (0.0%)	0 (0.0%)	
**Total positive**	**22 (37.3%)**	11 (18%)	5 (19%)	**16 (18.4%)**

NFS, nasopharyngeal swab; RS, rectal swab; C_T_, cycle threshold. ^*^10/11 toxigenic strains. Bold values are highlight the most relevant results and the total number of positive results.

**Table 5:** Serological tests and blood PCR for hepatitis performed in patients and pets.

Patients (*n* = 71)		
Serology		
*Toxocara canis* (IgG)	2/61 (3.2%)	
*Strongyloides* spp. (IgG)	9/61 (14.8%)	
*Toxoplasma gondii* (IgG)	0/21 (0.0%)	
*Bartonella* spp. (IgG)	4/21 (19%) *	
Hepatitis E virus (IgG)	4/71 (5.6%)	
Blood PCR HEV	0/71 (0.0%)	
**Total number of positives**	**16/71 (22.5%)**	
Pets (*n* =38)		
	Dogs (*n* = 31)	Cats (*n* = 7)
Serology		
*Leishmania* spp.	0/31 (0.0%)	**1/7 (14.3%)**
*Leptospira* spp. (in non-vaccinated pets)	0/31 (0.0%)	0/7 (0.0%)
*Borrelia burgdorferii*	0/31 (0.0%)	ND
*Rickettsia* spp.	**7/31 (22.6%)**	ND
*Ehrlichia canis*	0/31 (0.0%)	ND
*Babesia canis*	**2/31 (6.5%)**	ND
*Anaplasma* spp.	0/31 (0.0%)	ND
*Toxoplasma* (IgM)	ND	ND
*Toxoplasma* (IgG)	ND	1/7 (14.3%)**
Hepatitis E virus (IgG)	0/12 (0.0%)	0/5 (0.0%)
Blood PCR HEV	0/12 (0.0%)	0/5 (0.0%)
**Total number of positives**	**10/38 (26.3%)**	

PCR, polymerase chain reaction; HEV, hepatitis E virus; ND, not done. *In 2 of these cases, stray kittens were adopted during the 6 months after transplantation. **PCR for Toxoplasma gondii in stool samples was also performed in 10 cats, all with negative results. Bold values are highlight the most relevant results and the total number of positive results.

### Zoonosis risk analysis

We then analyzed the association between the presence of microorganisms and all hygiene and diet habits. These included the pet’s age, number of veterinary visits, deworming frequency and compliance with recommendations, type of food, outdoor activities, have seen the pet eat or hunt another animal, presence of ticks and adequate vaccination schedule. None of the variables analyzed was associated with a higher presence of microorganisms in pets and/or patients (Table 6).

**Table 6:** Relationship between pets’ and patients’ positive results and pets’ epidemiological data.

		Pets’ stool results		Pets’ serology		Patients’ serology	
		Positive	*p*-value	Positive	*p*-value	Positive	*p*-value
Pet age	<6 m	0.0% (0/16)	1	0.0% (0/7)	1	0.0% (0/16)	1
	>6 m	100.0% (16/16)		70.0% (7/10)		87.5% (14/16)	
Veterinarian visits^a^	Correct	81.2% (13/16)	0.3627	60.0% (6/10)	0.562	75.0% (12/16)	1
	Incorrect	18.7% (3/16)		10.0% (1/10)		12.5% (2/16)	
Internal deworming^b^	Correct	6.2% (1/16)	0.573	10.0% (1/10)	0.4	37.5% (6/16)	0.7837
	Incorrect	93.7% (15/16)		70.0% (7/10)		56.3% (9/16)	
External deworming	Yes	81.2% (13/16)	0.5385	70.0% (7/10)	0.5591	68.7% (11/16)	1
	No	18.8% (3/16)		0.0% (0/10)		18.8% (3/16)	
Feeding^c^	Correct	93.7% (15/16)	1	70.0% (7/10)	1	75.0% (12/16)	0.2131
	Incorrect	0.0% (0/16)		0.0% (0/10)		6.2% (1/16)	
Going outside	Yes	75.0% (12/16)	0.0934	30.0% (3/10)	0.3868	50.0% (8/16)	0.5391
	No	25.0% (4/16)		40.0% (4/10)		37.5% (6/16)	
Hunting	Yes	25.0% (4/16)	0.7538	0.0% (0/10)	0.5585	12.5% (2/16)	0.7134
	No	75%0.0 (12/16)		70.0% (7/10)		75.0% (12/16)	
Ticks	Yes	18.7% (3/16)	0.5406	10.0% (1/10)	0.3844	12.5% (2/16)	0.4844
	No	81.3% (13/16)		60.0% (6/10)		75.0% (12/16)	
Vaccination^d^	Correct	6.2% (1/16)	1	0.0% (0/10)	1	0.0% (0/16)	1
	Incorrect	62.5% (10/16)		70.0% (7/10)		100% (16/16)	

^a^Veterinarian visits were considered correct if they occurred at least once a year. ^b^Internal deworming was considered correct when it was performed at least once a month, following the recommendations of the ESCCAP guidelines for immunocompromised patients (23). ^c^Feeding was considered correct in case of processed or cooked food (not raw food). ^d^Vaccination was considered adequate in cases of dogs with vaccinations for rabies, *B. bronchiseptica* (injectable), *Leptospira*, and *Leishmania*; and in cases of cats with vaccinations for rabies and *B. bronchiseptica* (injectable). In our country (Spain) rabies virus vaccination is a core vaccine for cats and dogs and it is universally recommended, but it is not legally obligatory in all regions. Immunization with noncore vaccines should be considered in accordance with local recommendations: leptospirosis vaccination is recommended in certain areas because of the disease’s zoonotic nature and high pathogenicity; leishmaniosis vaccination and *Borrelia burgdorferi* vaccination are recommended for dogs living with immunocompromised individuals in endemic areas (although they would not prevent human disease); *Bordetella bronchiseptica* vaccination may be considered in situations of risk, such as doggy day-care centers, visits to the local dog park, and large colonies (inactivated vaccines should always be used in case of immunocompromised owners) (2). There were no statistically significant differences in any of the variables comparing the 2 groups.

## Discussion

This is one of the first studies, to our knowledge, assessing shared colonizations/infections in immunocompromised children and their pets aiming to analyze the role of dogs and cats as sources of zoonotic infections, including viral, bacterial and parasitic pathogens. Via a complete microbiological study, a noticeable number of microorganisms was identified in both patients and pets, with up to 44.6% of the patients and almost one-third of the pets testing positive for at least one microorganism under investigation. Although several potentially zoonotic agents were found in dogs and cats sharing a household with these patients, there was only one case of shared colonization (*Blastocystis*) and no zoonotic transmission could be demonstrated. Although gaps in preventive zoonotic measures were detected, no differences were found between pets with positive and negative zoonotic screening results and none of the studied factors was associated with a higher prevalence of colonization/infection among pets or children.

As described among human households (24), humans and pets can share microorganisms. However, the evidence remains scarce and the clinical implications are unknown. Correct deworming treatments in pets, adherence to scheduled immunization visits and following veterinary recommendations are strongly encouraged but might not cover the entire range of potential zoonotic pathogens that pets can harbor and the clinical impact in terms of zoonosis prevention has not been demonstrated (2). Immunocompromised hosts are more vulnerable to infections than their immunocompetent counterparts; therefore, the risks are presumably higher.

Our results reveal a high prevalence of pet ownership (47.9%), similar to previous data from our group (45.8%) (7) and from Europe (46%) (25). We found a considerable number of pathogens in our patients’ fecal samples (37.3%), whereas the number of pathogens in pets’ feces was lower (18.4%). Even so, potential zoonotic pathogens such as *Cryptosporidium* or *Campylobacter* were detected in pets’ feces. No helminths were found in pets’ feces, despite the high frequency of incorrect intestinal deworming practices (23) and considering that most of the pets were fed with commercially processed food. Clinicians should consider that routine deworming of pets involves anthelmintic drugs that are effective against cestodes and nematodes, but not against protists such as *Giardia* or *Cryptosporidium*. Previous molecular-based studies investigating the potential occurrence of zoonotic transmission events involving *Giardia* and *Cryptosporidium* among healthy individuals and their pets have failed to so demonstrate (26, 27). However, these surveys were hampered by transversal rather than longitudinal sampling designs and limited sample sizes; thus, to date, no previous studies, as well as our data, have demonstrated that these pathogens are a source of gastrointestinal cross-infections/colonizations.

*Giardia duodenalis* was detected in 5.6% of the studied patients in our cohort and in only 1.6% of the dogs. This is an unexpectedly low percentage, especially among pets, according to previously published data (26, 28–31). In Spain, previous epidemiological studies in the pediatric population have demonstrated the presence of *G. duodenalis* in 3–25% of asymptomatic children (32, 33). Among pets, the presence of *Giardia* in feces has been reported in 17.3–40.9% of owned and sheltered dogs (26, 28–31) and in 5.9 and 9.2% of owned and sheltered cats, respectively (26, 29). A potential explanation for this discrepancy is that families of immunocompromised children might be more aware of the risks associated with pet ownership and provide better care of their pets’ health compared with the general population. However, previous studies regarding pet ownership among immunocompromised young patients have revealed non-compliance with basic veterinary recommendations and risky exposures for acquiring zoonoses (7, 34). Most of our human and animal *Giardia*-positive samples yielded high (>35) cycle threshold (C_T_) values, indicative of low parasite loads. The only isolate successfully genotyped (assigned to zoonotic assemblage B) was identified in a human sample with a C_T_ value of 33.7.

Another remarkable parasite encountered was *Blastocystis* sp., which is probably the most common enteric parasite in humans globally (35), although its pathogenicity remains controversial (35). It was present in 6.8% of participants but only 1 dog. The role of companion animals as reservoirs of human *Blastocystis* infections is uncertain (35). A Spanish study conducted in Northern Spain found *Blastocystis* in 35.2% of the human stool samples analyzed, but not in any of the canine or feline fecal specimens investigated, suggesting that these pets play a negligible role as natural reservoirs of human *Blastocystis* infection (35). In our cohort, 4 human fecal samples were *Blastocystis*-positive. Three were successfully subtyped, allowing the identification of the subtypes ST1, ST2, and ST4, all common in European human populations. However, only one of the canine fecal samples tested positive for the parasite, although our molecular analyses failed to determine the subtype involved. *Blastocystis* was the only case of shared colonization (ST4 in a patient, unknown subtype in a dog) in our cohort. Therefore, pet dogs and cats do not appear to have a relevant role as reservoirs of human *Blastocystis* infections.

On the other hand, emerging pathogens are becoming increasingly relevant, such as *Enterocytozoon bieneusi*. This fungi-related pathogen is considered an emerging infectious agent, with the most common Microsporidia species contributing to human microsporidiosis; it is an opportunistic pathogen infecting immunocompromised individuals (36, 37). Some commonly reported human genotypes have been found in animals, raising the question of whether human-animal contact could play a role in its transmission to humans (12). *Enterocytozoon bieneusi* has been detected in 0.8% of owned dogs and 3% of owned cats in Northern Spain (38) and in 0.4% of dogs in the Madrid area (Central Spain) (30). In our series, the prevalence of *E. bieneusi* in dogs and cats was 3.3 and 0.0%, respectively, and there were no positive results in humans. In addition, *Encephalitozoon intestinalis* DNA was identified in a feline fecal sample. This is the first report of the presence of *E. intestinalis* in this host in Spain. Taken together, these data indicate that companion animals might act as a potential source of human microsporidiosis.

Regarding bacterial findings in feces, no *C. difficile* isolates were identified in our canine and feline populations, although the owners were highly colonized, probably due to a high number of previous hospitalizations and frequent use of antibiotherapy. A recent review of several studies in various countries worldwide on the prevalence and molecular epidemiology of *C. difficile* in dogs and cats revealed variable colonization rates (39). In healthy dogs, the colonization rate was shown to be 3–5.5% and this percentage increased to 12% in dogs with gastrointestinal diseases. Similarly, *C. difficile* was isolated in 2.5–9.4% of healthy and diarrheal cats (39). These studies have shown that pets carry strains genetically identical to that of their owners, suggesting inter-species transmission (39, 40). Similarly to our results, few pets were infected in a recent small prospective study conducted in the USA in patients with diarrhea and their pets (owned dogs and cats) (40). Only in 2 households was *C. difficile* detected in both the owner and pet, although these strains were different (40). Two studies conducted in veterinary clinics from the Madrid region (Central Spain) reported prevalence of *C. difficile* in feces from owned dogs and owned cats of 4.8 and 0.0%, respectively (41), and of 6.7% in diarrheic dogs (42). These data suggest low probability of cross-transmission.

Taking into account serological tests, our results show previous exposure to several zoonotic agents in both patients and pets.

*Strongyloides stercoralis* can lead to severe hyperinfection and disseminated strongyloidiasis in immunocompromised patients (43). Its prevalence in this specific clinical population is not well documented and recent studies have reported prevalence rates of approximately 3–5% (43). It should be noted that reported prevalence rates were based on a limited number of heterogeneous studies that differ in the study regions and the diagnostic methods used (43). The results from our patients are in contrast to those published by other authors, with a much higher *Strongyloides* seroprevalence (14.8%). Although all infected patients in our cohort lived with dogs, the patients could have been infected by walking barefoot or by playing with soil (44). To date, it remains unclear whether dogs act as a suitable reservoir for human infections.

Toxocariasis is another neglected zoonotic infection, dogs and cats being the natural definitive hosts (45). Given that the majority of the infected individuals remain asymptomatic (45), its prevalence can be underestimated. In a previous study conducted in our center, we found a seroprevalence for toxocariasis of 5.3% among migrant and internationally adopted children (45). In our series, 2 asymptomatic patients were seropositive (3.2%). However, severe forms such as ocular or cerebral toxocariasis could occur in immunocompromised hosts (46); thus, screening based on serology should be performed in immunocompromised patients.

*Bartonella henseale* poses a notable risk to cat owners. Infected individuals may experience symptoms such as cat scratch disease, fever, lymphadenopathy, fatigue or muscle pain. While the infection typically remains mild, severe complications such as pulmonary nodules, pneumonia, ocular and skin lesions, osteomyelitis, hepatosplenic disease, bacillary angiomatosis or encephalitis can occur, especially in immunocompromised individuals (2). Up to 19% (4/21) of positive results for *Bartonella henselae* were observed among cat owners. Interestingly, half of these positive patients (*n* = 2) adopted stray cats a few months after transplantation, confirming important gaps in zoonotic risk knowledge in this population (7). In our country, the prevalence of *Bartonella* varies between series: 8.7% in healthy people from Catalonia (Northern Spain) (47), 22.3% among patients with HIV from the same region (48) and up to 37.1% among the veterinary worker population (49). Specifically in cat owners, a seroprevalence of 6.07% was estimated in a Chilean study (50), lower than our results.

None of the cats’ stools tested positive for *Toxoplasma gondii* and no patient had a positive serology. Although toxoplasmosis has traditionally been linked to contact with cats, the majority of infections in Europe occur by other means of transmission (51). A recent meta-analysis has observed that although the pooled prevalence of oocysts in European domestic cats’ feces is as low as 1.2%, their presence in soil is found in up to 16% (52). The risk is extremely low for indoor urban domestic cats (52). These findings highlight the lack of evidence supporting most recommendations to prevent zoonoses.

Many families (23.9%) reported having found ticks on their pets and a relevant percentage of dogs (29.0%) presented positive serology for microorganisms such as *Rickettsia* spp. or *Babesia canis*. A previous Chilean study revealed the presence of ectoparasites in nearly 50% of dogs and cats (3). Ticks can be vectors of serious infections in the USA and in Europe, such as Lyme disease, borreliosis, Central European encephalitis, or Crimean Congo hemorrhagic fever. The geographical distribution of this tick species has been expanding and an increase in tick-borne infections has recently been reported (53–55). Curiously, although all dogs tested were negative, one case of positive serology for *Leishmania* was detected in a cat. A recent study performed in our country found that 2% of stray cats were seropositive for *Leishmania* (56); thus, although infrequent, these felines could also be infected.

HEV and ratHEV (*Rocahepevirus ratti*) are 2 emerging viruses affecting humans for which cats and dogs might serve as hosts, as shown in previous studies (11, 57). A study in southern Spain reported a prevalence of anti-HEV antibodies in dogs and cats of 10 and 2.8%, respectively (11), suggesting that these species might play a potential role in the HEV zoonotic cycle. Similarly, this study provides evidence of ratHEV circulation in these species, indicating that cats and dogs might serve as reservoirs. This potential susceptibility was confirmed in a study conducted in Hong Kong, which reported that 1.2% of dogs and 1.5% of cats in the area exhibited IgG antibodies against ratHEV (57). However, the risk of zoonotic transmission from pets to humans was deemed minimal, given that none of the studies found evidence of viral RNA. Our study is the first to report the presence of HEV in these species (feces from 1 dog and 1 cat), both of which harbored strains capable of zoonotic transmission, such as HEV-3 f. Similarly, we report for the first time the presence of ratHEV in cats and dogs, suggesting that these species could also be susceptible to infection by this recently described zoonotic virus. Although the source of HEV infection cannot be definitively identified, the most plausible route could be through the consumption of raw or undercooked meat, because it constitutes the most efficient transmission pathway. In the case of ratHEV, although the primary host of this virus appears to be rodents, the route of infection between animal species and zoonotic transmission remains unknown. In fact, the dog in our sample with ratHEV identified in stool samples consumed raw or undercooked food on a monthly basis. The absence of infection in the children owning these animals reinforces the idea that the risk of transmission from these species through direct contact could be minimal, and thus, they are likely play a limited role in the epidemiology of these viruses.

Bidirectional bacterial transmission between owners and pets has already been reported (24, 58). According to a previous study by our group, up to 16% of children with complex chronic conditions are *S. aureus* colonized, with up to 27% of them colonized by multidrug-resistant *Enterobacteriaceae* (59). We hypothesize that pets living with immunocompromised children might be more frequently colonized by multidrug-resistant pathogens, and that pets could act as reservoirs, maintaining transmission in the community. However, the unexpectedly low colonization rates observed in our patients and pets did not allow us to observe possible cross-colonization. The prevalence of *S. pseudintermedius* colonization in the nasopharyngeal swabs (12%) of the screened dogs was also lower than expected; previous studies have reported colonization rates in dogs from 43 to 92% (13). Dogs can be persistent or intermittent carriers, so collecting more than one sample at various time points could have increased our ability to detect colonizations (13, 60).

Despite the high number of global positive results among both pets and patients, we found no association with pet age, veterinary visits, vaccination, deworming, hunting, presence of ticks, or feeding compared with the pets with negative results. However, the small sample size has limited the analysis. Nonetheless, we detected a few interesting findings related to zoonosis risk, such as stray cat ownership a few months after transplantation in half of the children with positive serology for *Bartonella*, or the consumption of raw or undercooked meat in one dog with ratHEV identified in stool samples. These findings are indicative of important gaps in zoonotic risk knowledge among this vulnerable population.

Some 77% of the surveyed patients considered pet ownership a benefit. Facing a life-threatening condition requiring long-term treatment has significant emotional implications and animal contact can offer substantial mental health benefits (2). Taking into account our results and considering that most of these zoonoses could be prevented, the balance between the psychological benefits and health risks for these patients appears to lean in favor of benefits, as long as basic veterinary recommendations are followed. However, our findings have limitations and deserve cautious interpretation. Close collaboration between veterinary and medical doctors as well as an enhanced role of veterinarians is required and patients should receive evidence-based information (8).

Our study has several limitations. It was a single-center study; thus, the number of patients and pets analyzed is relatively low and it might not be generalizable to other populations. Patient recruitment was complex and not all samples were collected for all participants, especially those from pets. In addition, its transversal design and the lower than expected number of individuals colonized impaired the identification of shared colonizations and/or zoonotic transmission events in our series. Samples were collected at a single time point; thus, zoonosis transmission could not be demonstrated.

## Conclusion

This is one of the first studies addressing the presence of colonization and zoonotic infections among immunocompromised children and their pets. We found that many pets living with immunocompromised children are infected by zoonotic pathogens and we observed previous exposure to zoonotic agents in both patients and pets. However, shared colonization was rare and could not be explained by diet/hygiene habits; thus, larger studies are warranted in order to address the role of pets as zoonosis reservoirs. In the meantime, our data are reassuring, because no additional risk was identified for immunocompromised children having pets (dogs and/or cats). Given that pets have important socio-emotional benefits, defining the potential risks and effective preventive interventions is very much needed to increase the quality of life of immunocompromised patients.

## Data availability statement

The original contributions presented in the study are included in the article/Supplementary material, further inquiries can be directed to the corresponding author.

## Ethics statement

The studies involving humans and animals were approved by Clinical Research Ethics Committee of La Paz University Hospital (PI-4770). The studies were conducted in accordance with the local legislation and institutional requirements. Written informed consent for participation in this study was provided by the participants' legal guardians/next of kin. Written informed consent was obtained from the owners for the participation of their animals in this study.

## Author contributions

PG-S: Conceptualization, Data curation, Formal analysis, Investigation, Methodology, Writing – original draft, Writing – review & editing. DR-T: Conceptualization, Data curation, Formal analysis, Investigation, Methodology, Writing – review & editing. IF-R: Data curation, Resources, Writing – review & editing, Methodology. PN: Data curation, Resources, Writing – review & editing, Methodology. GR-C: Conceptualization, Data curation, Resources, Writing – review & editing, Methodology. DC: Data curation, Resources, Supervision, Writing – review & editing, Methodology. MC: Data curation, Investigation, Resources, Writing – review & editing, Methodology. AR-J: Data curation, Investigation, Resources, Writing – review & editing, Methodology. LM: Conceptualization, Data curation, Methodology, Resources, Writing – review & editing. JR: Data curation, Investigation, Methodology, Resources, Writing – review & editing. FE: Investigation, Resources, Writing – review & editing, Data curation. BP-H: Conceptualization, Data curation, Formal analysis, Investigation, Methodology, Writing – review & editing. RS-L: Data curation, Investigation, Methodology, Writing – review & editing. JH-G: Data curation, Investigation, Methodology, Writing – review & editing. SA: Conceptualization, Data curation, Formal analysis, Investigation, Software, Writing – review & editing. TS: Supervision, Writing – review & editing, Formal analysis. CC: Supervision, Writing – review & editing, Formal analysis. AM-E: Conceptualization, Funding acquisition, Investigation, Methodology, Resources, Supervision, Writing – review & editing, Formal analysis.
