# Supplementary material for: Zoonosis screening in Spanish immunocompromised children and their pets
Source: Front Vet Sci. 2024 Jul 23;11:1425870. doi: 10.3389/fvets.2024.1425870 (PMC11300328; doi:10.3389/fvets.2024.1425870)
Supplement: Supplementary file 1 [file Data_Sheet_1.DOCX]

# **Supplementary file 1.** *Surveys for collecting pets´ and patients’ clinical data, and patients perceptions regarding pets´ ownership.*

# **SURVEY FOR PATIENTS**

Patient code:_______________

Survey date: ________________

Date of birth:_______________

Gender

- Male
- Female
- I prefer not to answer

Year of transplantation/diagnosis of disease: ___________

Type of transplantation:

- Intestinal transplantation
- Liver transplantation
- Renal transplantation
- Heart transplantation
- Lung transplantation
- Multivisceral transplantation
- Bone marrow transplantation
- None

Is the patient receiving chemotherapy or immunosuppressive treatment?

- Yes
- No

Do you currently own a pet?

- Yes
- No

What pet do you currently have? ___________

In my case, despite my illness, I believe that the benefit of having a pet outweighs the risk:

- Yes
- No

# **SURVEY FOR COLLECTING DOGS’ DATA**

Dog code: _____

Year when the dog started living with the patient: ___________

Age of the dog when it started to live with the patient:

- <6 months
- 6 months – 1 year
- 1 year – 5 years
- >5 years

Number of veterinary visits:

- <1 time/year
- 1 time/year
- 2 times/year
- ≥3 times/year

Does your pet receive intestinal deworming?

- Yes
- No

If you answered “yes” to the previous question, specify the frequency of intestinal deworming:

- Once a month
- Every three months
- Every six months
- Sporadically

Is your pet treated for external deworming?

- Yes
- No

If you answered “yes” to the previous question, specify the frequency of external deworming:

- Once a month
- Every 3 months
- Every 6 months
- Sporadically

Indicate the type of food your pet eats more frequently:

- Commercial processed food
- Home cooked food
- Raw or undercooked homemade food

Do you follow your veterinarian's recommendations regarding the frequency of vaccinations for your dog?

- Yes
- No

Does your pet frequently go outdoors or have contact with other animals outdoors?

- Yes
- No

If you answered “yes” to the previous question, please indicate the frequency:

- Daily
- 1 time/week
- Monthly
- Every 6 months
- 1 time/year

Have you ever seen your pet eat or hunt another animal?

- Yes
- No

Has your dog ever had ticks?

- Yes
- No

Has your dog ever been diagnosed with an infectious disease?

- Yes
- No

Is your dog vaccinated against rabies?

- Yes
- No
- I do not know

Is your dog vaccinated against *Bordetella bronchiseptica*?

- Yes
- No
- I do not know

Indicate the type of vaccine against *B. bronchiseptica*:

- Nasal
- Oral
- Injectable
- I do not know

Does your dog have any preventive measures against leishmania?

- Vaccine
- Collar
- Pipette
- None

Is your dog vaccinated against *Leptospira*?

- Yes
- No
- I do not know

# **SURVEY FOR COLLECTING CATS’ DATA**

Cat code: _____

Year when the cat started living with the patient: ___________

Age of the cat when it started to live with the patient:

- <6 months
- 6 months – 1 year
- 1 year – 5 years
- >5 years

Number of veterinary visits:

- <1 time/year
- 1 time/year
- 2 times/year
- ≥3 times/year

Does your pet receive intestinal deworming?

- Yes
- No

If you answered “yes” to the previous question, specify the frequency of intestinal deworming:

- Once a month
- Every 3 months
- Every 6 months
- Sporadically

Is your pet treated for external deworming?

- Yes
- No

If you answered “yes” to the previous question, specify the frequency of external deworming:

- Once a month
- Every 3 months
- Every 6 months
- Sporadically

Indicate the type of food your pet eats more frequently:

- Commercial processed food
- Home cooked food
- Raw or undercooked homemade food

How often does your cat eat raw or undercooked food?

- Once a month
- Every six months
- Once a year
- Never

Do you follow your veterinarian's recommendations regarding the frequency of vaccinations for your cat?

- Yes
- No

Does your pet frequently go outdoors or have contact with other animals outdoors?

- Yes
- No

If you answered “yes” to the previous question, please indicate the frequency:

- Daily
- 1 time/week
- Monthly
- Every 6 months
- 1 time/year

Have you ever seen your pet eat or hunt another animal?

- Yes
- No

Has your cat ever had ticks?

- Yes
- No

Has your cat ever been diagnosed with an infectious disease?

- Yes
- No

If you answered “yes” to the previous question, please specify the disease and year of diagnosis: ____________________

Is your cat vaccinated against rabies?

- Yes
- No
- I do not know

Is your cat vaccinated against *Bordetella bronchiseptica*?

- Yes
- No
- I do not know

Indicate the type of vaccine against *B. bronchiseptica*:

- Nasal
- Oral
- Injectable
- I do not know
