# Supplementary material for: Zoonosis screening in Spanish immunocompromised children and their pets
Source: Front Vet Sci. 2024 Jul 23;11:1425870. doi: 10.3389/fvets.2024.1425870 (PMC11300328; doi:10.3389/fvets.2024.1425870)
Supplement: Supplementary file 2 [file Data_Sheet_2.docx]

***Supplementary file 2.*** *Details of microbiological tests and specific kits used*

**Stool culture and PCR for fecal pathogens:** First, a stool culture and multiplex polymerase chain reaction (PCR) were performed with *Allplex™ Gastrointestinal Panel Assays^®^: Allplex™ GI-Bacteria (I) and Allplex™ GI-Parasite* (1) in both patients and pets. After these, stool samples were again tested for the following pathogens, which were genotyped:

- *Giardia duodenalis* DNA detection was achieved using a real-time PCR (qPCR) method, targeting the gene codifying the small subunit ribosomal RNA (*ssu* rRNA) of the parasite (2). The molecular diversity of the parasite at the assemblage level was investigated only in the *Giardia* isolates that tested positive by qPCR and yielded cycle threshold (C_T_) values ≤34. To do so, a nested PCR was employed to amplify a fragment of the *ssu* rRNA gene (3,4).
- The presence of *Cryptosporidium* spp. was assessed with a nested-PCR protocol to amplify a fragment of the *ssu* rRNA gene of the parasite (5).
- Identification of *Blastocystis* spp. was achieved by a direct PCR protocol targeting a fragment of the *ssu* rRNA gene of the parasite (6).
- *Enterocytozoon* *bieneusi* detection was conducted by a nested PCR protocol to amplify a fragment of the internal transcribed spacer (ITS) region as well as portions of the flanking large and small subunit of the ribosomal RNA gene as previously described (7).
- Detection of *Encephalitozoon* genus members (*E. intestinalis*, *E. cuniculus*, *E. hellem*) was conducted with a direct PCR protocol to amplify an *ssu* rRNA gene fragment of the parasite (8).
- PCR assays targeting 2 different and conserved regions of the viral genome for the diagnosis of acute hepatitis E virus (HEV) infection as well as molecular evaluation of HEV and ratHEV  were performed (9,10,11).
- Real-time PCR testing for *Toxoplasma gondii* in feces from cats and *Leishmania* spp. in conjunctival swabs from dogs: Total nucleic acid (DNA and RNA) was extracted from 0.2 g feces or 200 µl swab eluate, respectively, with the "MagMAX™ CORE Nucleic Acid Purification Kit" (ThermoFisher) via the KingFisher Flex platform (ThermoFisher), according to the manufacturer’s instructions. Total nucleic acid was eluted in 200 µl (feces) or 100 µl (swab) of elution buffer (ThermoFisher), and 5 µl was amplified in subsequent single plex real-time PCR reactions. Real-time PCR was performed at IDEXX, Vet Med Labor GmbH, employing the LightCycler 480 system (Roche) with proprietary forward and reverse primers and hydrolysis probes. The target gene for *Toxoplasma gondii* detection was the internal transcribed spacer 1 (ITS-1) gene (L49390), and for *Leishmania* spp. Detection it was the major surface glycoprotein gene, GP63 (Y08156).
- For the detection helminths, a multiplex fluorescence immunoassay method detecting antigens of Ancylostomatidae, Ascarididae, Trichuridae, Dipylidium caninum, and Giardia were performed, only in feline and canine feces (Idexx Fecal Dx®)(12,13).

**PCR assays performed in blood**: PCR assays for the diagnosis of acute hepatitis E virus (HEV) infection, as well as molecular evaluation of HEV and ratHEV, were performed on patients’ and pets’ blood (9,10,11). In dogs and cats, a PCR for detecting *Leishmania* spp. was also performed (Idexx).

**Serology:** Several serological tests were performed in patients and pets, which are described in *Table S3.*

**Nasopharyngeal and rectal swabs:** In both patients and pets, we performed nasopharyngeal swab cultures for the detection of *Staphylococcus aureus* and *S. pseudintermedius* colonization. In addition, rectal swabs were performed for the detection of extended-spectrum beta-lactamase-producing *Enterobacteriaceae* and the carbapenem-resistant bacteria *Enterobacteriaceae*.

***TABLE S1.*** *Polymerase chain reaction protocols used for the molecular identification and/or characterization of protist pathogens in the human and animal fecal samples investigated in this study*

|  |  | **Temperature and time** | | | |  |  |  |
| --- | --- | --- | --- | --- | --- | --- | --- | --- |
| **Target organism** | **Locus** | **Initial denaturation** | **Denaturation** | **Annealing** | **Extension** | **No. cycles** | **Final extension** | **Reference** |
| *Giardia duodenalis* | *ssu* rRNA | 95 °C 15 min | 95 °C 15 s | 60 °C 1 min | 72 °C 30 s | 45 | – | Verweij et al., 2003 (2) |
|  | *ssu* rRNA | 95 °C 2 min | 95 °C 45 s | 58/55 °C 30 s | 72 °C 45 s | 35 | 72 °C 4 min | Appelbee et al., 2003 (3) |
| *Cryptosporidium* spp. | *ssu* rRNA | 94 °C 3 min | 94 °C 40 s | 50 °C 40 s | 72 °C 1 min | 35 | 72 °C 10 min | Tiangtip and Jongwutiwes, 2002 (5) |
| *Blastocystis* spp. | *ssu* rRNA | 95 ºC 3 min | 94 ºC 1 min | 59 ºC 1 min | 72 ºC 1 min | 30 | 72 °C 2 min | Scicluna et al., 2006 (6) |
| *Enterocytozoon bieneusi* | ITS | 94 ºC 3 min | 94 ºC 30 s | 57/55 ºC 30 s | 72 ºC 40 s | 35 | 72 ºC 10 min | Buckholt et al., 2002 (7) |
| *Encephalitozoon* spp. | *ssu* rRNA | 94 °C 10 min | 94 °C 30 s | 60 °C 30 s | 72 °C 30 s | 35 | 72 ºC 10 min | Fedorko et al., 1995 (8) |

***TABLE S2.*** *Oligonucleotides used for the molecular identification and/or characterization of the protist pathogens in animal fecal samples investigated in this study*

| **Target organism** | **Locus** | **Oligonucleotide** | **Sequence (5´–3´)** | **Generated amplicon**  **(bp)** | **Reference** |
| --- | --- | --- | --- | --- | --- |
| *Giardia duodenalis* | *ssu* rRNA | Probe | FAM–CCCGCGGCGGTCCCTGCTAG–BHQ1 | 62 | Verweij et al., 2003 (2) |
|  |  | Gd-80F | GACGGCTCAGGACAACGGTT |  |  |
|  |  | Gd-127R | TTGCCAGCGGTGTCCG |  |  |
|  | *ssu* rRNA | Gia2029 | AAGTGTGGTGCAGACGGACTC | 300 | Appelbee et al; 2003 (3) |
|  |  | Gia2150c | CTGCTGCCGTCCTTGGATGT |  |  |
|  |  | RH11 | CATCCGGTCGATCCTGCC |  | Hopkins et al., 1997 (4) |
|  |  | RH4 | AGTCGAACCCTGATTCTCCGCCAGG |  |  |
| *Cryptosporidium* spp. | *ssu* rRNA | CR-P1 | CAGGGAGGTAGTGACAAGAA | 587 | Tiangtip and Jongwutiwes, 2002 (5) |
|  |  | CR-P2 | TCAGCCTTGCGACCATACTC |  |  |
|  |  | CR-P3 | ATTGGAGGGCAAGTCTGGTG |  |  |
|  |  | CPB-DIAGR | TAAGGTGCTGAAGGAGTAAGG |  |  |
| *Blastocystis* spp. | *ssu* rRNA | BhRDr | GAGCTTTTTAACTGCAACAACG | 600 | Scicluna et al., 2006 (6) |
|  |  | RD5 | ATCTGGTTGATCCTGCCAGT |  |  |
| *Enterocytozoon bieneusi* | ITS | EBITS3 | GGTCATAGGGATGAAGAG | 390 | Buckholt et al., 2002 (7) |
|  |  | EBITS4 | TTCGAGTTCTTTCGCGCTC |  |  |
|  |  | EBITS1 | GCTCTGAATATCTATGGCT |  |  |
|  |  | EBITS2.4 | ATCGCCGACGGATCCAAGTG |  |  |
| *Encephalitozoon* spp. | *ssu* rRNA | MICROF | CACCAGGTTGATTCTGCCTGA | 250–270 | Fedorko et al., 1995 (8) |
|  |  | MICROR | CCTCTCCGGAACCAAACCCTG |  |  |

*ITS: internal transcribed spacer; ssu rRNA: small subunit ribosomal RNA*

***TABLE S3.*** *Serological tests performed in patients and pets*

| **PETS’ SEROLOGY** | | | |
| --- | --- | --- | --- |
|  | | **Method** | **Kit** |
| **Dog** | *Leishmania* (Ab) | ELISA | CIVTEST Leishmania, Hipra |
|  | *Ehrlichia canis* (Ab) | ELISA | VetLine Ehlichia, NovaTec Inmunodiagnostica |
|  | *Borrelia burgdorferi* (Ab) | ELISA | SNAP 4DX Plus IDEXX |
|  | *Rickettsia* spp. (Ab) | IFI | MegaFLUO Rickettsia, Megacor |
|  | *Babesia canis* (Ab) | ELISA | BABESIA-ELISA DOG, AFOSA |
|  | *Anaplasma* spp. (Ab) | ELISA | SNAP 4DX Plus IDEXX |
|  | *Leptospira* (Ab) | MAT | MAT was performed according to OIE standards (Office International des Epizooties OIE, 2008) |
| **Cat** | *Leishmania* (Ab) | IFI | MegaFLUO LEISH kit, Megacor |
|  | *Leptospira* (Ab) | MAT | MAT was performed according to OIE standards (Office International des Epizooties OIE, 2008) |
|  | *Toxoplasma* IgM (Ab) | IFI | Anti-Toxoplasma gondii IIFT Dog (IgM), Euroinmun |
|  | *Toxoplasma* IgG (Ab) | IFI | Anti-Toxoplasma gondii IIFT Dog (IgG), Euroinmun |
| **PATIENTS’ SEROLOGY** | | | |
|  | | **Method** | **Kit** |
| **Patients owning dogs** | *Toxocara canis* (IgG) | ELISA | NovaLisa Toxocara canis IgG (Novatec) |
|  | *Strongyloides* (IgG) | ELISA | NovaLisa Strongyloides IgG (Novatec) |
|  | *Hepatitis E virus* | ELISA | Wantai Diagnostic IgG kit (Beijing, China) |
| **Patients owning cats** | *Bartonella henselae* (IgG) | CLIA | Bartonella henselae Virclia IgG MONOTEST (Vircell) |
|  | *Toxoplasma gondii* (IgG) | CLIA | *Toxoplasma gondii* Virclia IgG MONOTEST (Vircell) |
|  | *Hepatitis E virus* | ELISA | Wantai Diagnostic IgG kit (Beijing, China) |

*Ab: antibodies; CLIA: chemiluminescence immunoassay; ELISA: enzyme-linked immunosorbent assay; IFI: indirect immunofluorescence; MAT: microagglutination test; Ag: antigen.*

**References**

1. Ligero-López J, García-Rodríguez J, Ruiz-Carrascoso G. Diagnosis of gastrointestinal infections: Comparison between traditional microbiology and a commercial syndromic molecular-based panel. *FEMS Microbiol Lett*. (2023) *370*:fnad122. doi: 10.1093/femsle/fnad122
2. Verweij JJ, Schinkel J, Laeijendecker D, van Rooyen MA, van Lieshout L, Polderman AM. Real-time PCR for the detection of *Giardia lamblia*. *Mol Cell Probes.* (2003) 17:223-5. doi: 10.1016/s0890-8508(03)00057-4
3. Appelbee AJ, Frederick LM, Heitman TL, Olson ME. Prevalence and genotyping of *Giardia duodenalis* from beef calves in Alberta, Canada. *Vet Parasitol*. (2003) 112:289-94. doi: 10.1016/s0304-4017(02)00422-3
4. Hopkins RM, Meloni BP, Groth DM, Wetherall JD, Reynoldson JA, Thompson RC. Ribosomal RNA sequencing reveals differences between the genotypes of *Giardia* isolates recovered from humans and dogs living in the same locality. *J Parasitol*. (1997) 83:44-51
5. Tiangtip R, Jongwutiwes S. Molecular analysis of *Cryptosporidium* species isolated from HIV-infected patients in Thailand. *Trop Med Int Health.* (2002) 7:357-64. doi: 10.1046/j.1365-3156.2002.00855.x
6. Scicluna SM, Tawari B, Clark CG. DNA barcoding of *Blastocystis*. *Protist.* (2006) 157:77-85. doi: 10.1016/j.protis.2005.12.001
7. Buckholt MA, Lee JH, Tzipori S. Prevalence of *Enterocytozoon bieneusi* in swine: an 18-month survey at a slaughterhouse in Massachusetts. *Appl Environ Microbiol.* (2002) 68:2595-9. doi: 10.1128/AEM.68.5.2595-2599.2002
8. Fedorko DP, Nelson NA, Cartwright CP. Identification of microsporidia in stool specimens by using PCR and restriction endonucleases. *J Clin Microbiol.* (1995) 33:1739-41. doi: 10.1128/jcm.33.7.1739-1741.1995
9. Lopez-Lopez P, Frias M, Perez-Jimenez AB, Freyre-Carrillo C, Pineda JA, Aguilera A, *et al*. Optimization of the molecular diagnosis of the acute hepatitis E virus infection. *Microb Biotechnol*. (2023) *16*:1325-32. doi: 10.1111/1751-7915.14247
10. Casares-Jimenez M, Rivero-Juarez A, Lopez-Lopez P, Montes ML, Navarro-Soler R, Peraire J, *et al*. Rat hepatitis E virus (Rocahepevirus ratti) in people living with HIV. *Emerg Microbes Infect*. (2024) 13:2295389. doi: 10.1080/22221751.2023.2295389
11. Frías M, López-López P, Zafra I, Caballero-Gómez J, Machuca I, Camacho A, *et al*. Development and Clinical Validation of a Pangenotypic PCR-Based Assay for the Detection and Quantification of Hepatitis E Virus (Orthohepevirus A Genus). *J Clin Microbiol*. (2021) 59:e02075-20. doi: 10.1128/JCM.02075-20
12. Elsemore D, Bezold T, Geng J, Hanna R, Tyrrell P, Beall M. Immunoassay for detection of *Dipylidium caninum* coproantigen in dogs and cats. *J Vet Diagn Invest.* (2023) *35*: 671-78. [doi: 10.1177/10406387231189193](https://doi.org/10.1177/10406387231189193)
13. Elsemore, DA. Antigen detection: Insights into *Toxocara* and other ascarid infections in dogs and cats. *Adv Parasitol*. (2020) 109, 545-59. [doi: 10.1016/bs.apar.2020.01.034](https://doi.org/10.1016/bs.apar.2020.01.034)
