# Supplementary material for: Zoonosis screening in Spanish immunocompromised children and their pets
Source: Front Vet Sci. 2024 Jul 23;11:1425870. doi: 10.3389/fvets.2024.1425870 (PMC11300328; doi:10.3389/fvets.2024.1425870)
Supplement: Supplementary file 3 [file Data_Sheet_3.DOCX]

***Supplementary file 3.*** *Specific molecular data and sequencing of pathogens from feces*

|  | **Molecular details** | **Human/pet** | **Additional comments** |
| --- | --- | --- | --- |
| ***Blastocystis* spp.**  (Subtypes and alleles) | ST1, allele 4 | 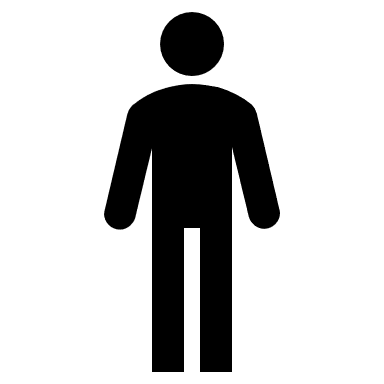 | ST1-ST4 are the usual human subtypes. *Blastocystis* spp. is a rare finding in strict carnivores, so it is infrequently found in animals with a mainly carnivorous diet such as dogs or cats. |
|  | ST2, allele 12 | 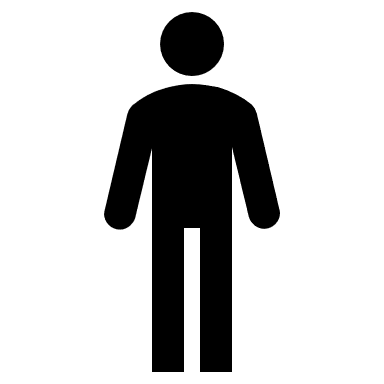 |  |
|  | ST4, allele 42 | 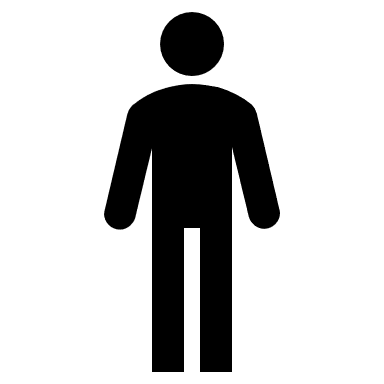 |  |
| ***Encephalitozoon* spp*.***  (Sequencing) | *E. intestinalis* | 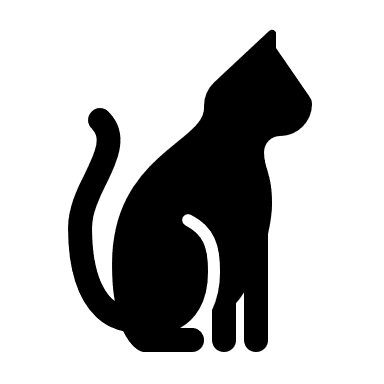 | First report of *E. intestinalis* in cats in Spain |
| ***Enterocytozoon bieneusi*** (Sequencing) | PtEbIX | 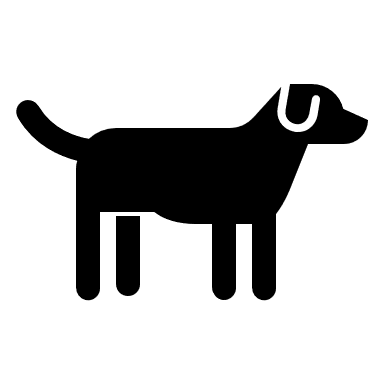 | PtEbIX is a canine-adapted genotype with no zoonotic potential |
|  | PtEbIX | 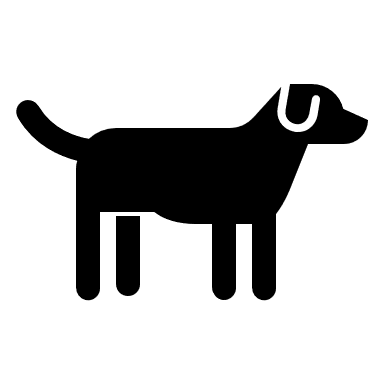 |  |
|  | PtEbIX | 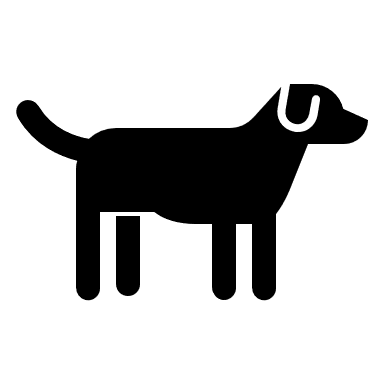 |  |
| ***Giardia duodenalis***  (C_T_ and sequencing) | 38.5, unknown assemblage | 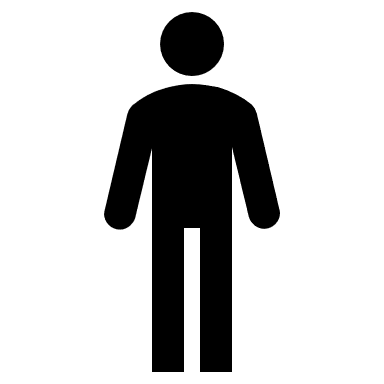 | Isolates with high C_T_ values at real-time polymerase chain reaction indicate low parasite loads and are untypable |
|  | 37.6, unknown assemblage | 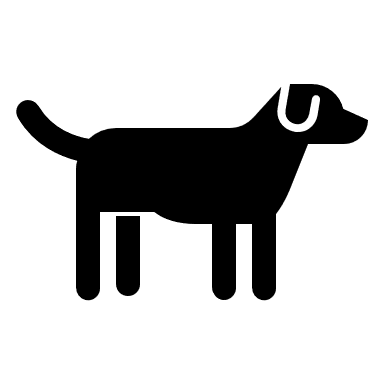 |  |
|  | 36.9, unknown assemblage | 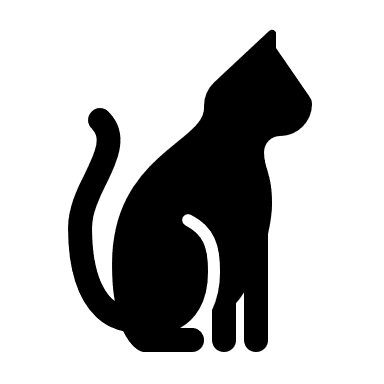 |  |
|  | 35.6, unknown assemblage | 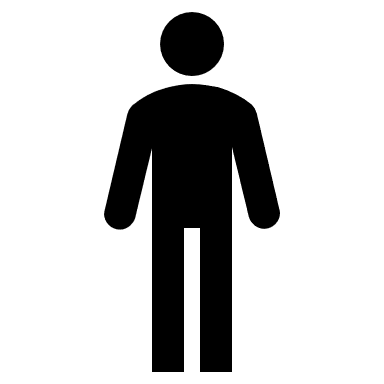 |  |
|  | 33.7, assemblage B | 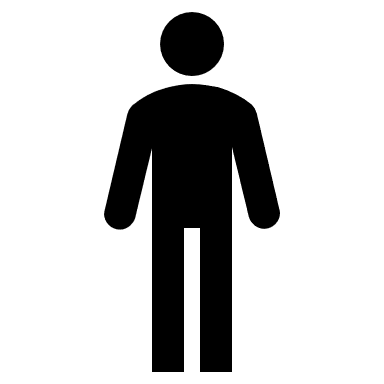 | Zoonotic genotype |
| ***Hepevirus***  (Sequencing) | HEV-3f | 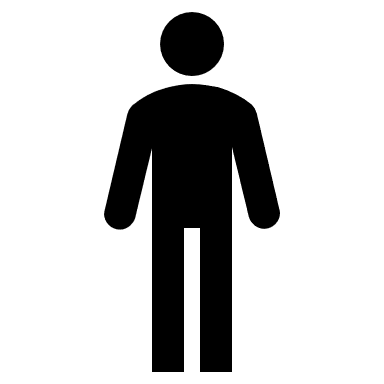 | Zoonotic genotype |
|  | HEV-3f | 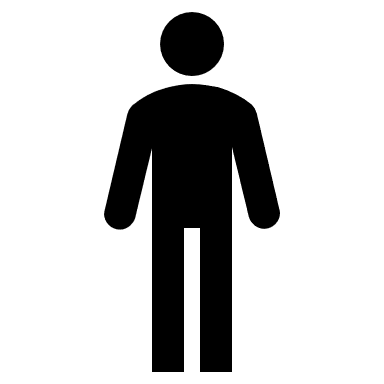 |  |
|  | HEV-3f | 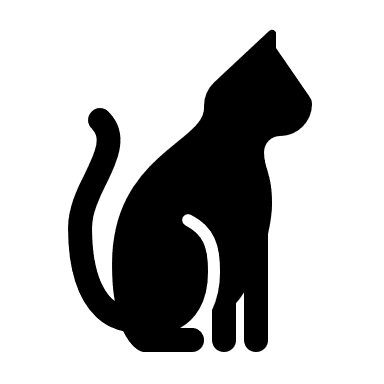 |  |
|  | RHEV (*Rocahepevirus ratti*) | 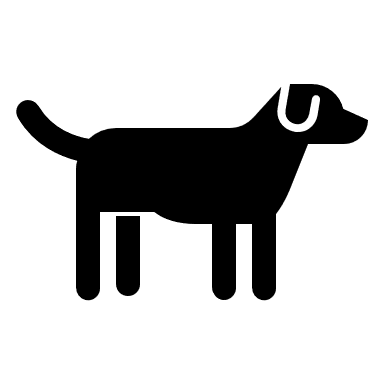 | Zoonotic genotype  The dog's diet consisted mainly of processed commercial food; however, on a monthly basis the dog consumed raw/undercooked food. |

*C_T_: Cycle threshold*
